# Enhancement of antibiotic therapy in osteomyelitis by inhibiting talin-1-mediated bacterial internalization

**DOI:** 10.3389/fcimb.2026.1823065

**Published:** 2026-08-06

**Authors:** Yuntao Li, Shanliang Jin, Hairong Tao

**Affiliations:** 1 Department of Orthopedics, Shanghai Key Laboratory of Orthopedic Implant, Shanghai Ninth People’s Hospital, Shanghai Jiao Tong University School of Medicine, Shanghai, China; 2 Department of Anesthesia, Shanghai Ninth People’s Hospital, Shanghai Jiao Tong University School of Medicine, Shanghai, China

**Keywords:** antibiotic, bacterial internalization, osteomyelitis, *Staphylococcus aureus*, talin-1

## Abstract

Intracellular infection by *Staphylococcus aureus* (*S. aureus*) is a major driver of persistent osteomyelitis, leading to antibiotic failure and recurrent bone destruction. However, the precise host-pathogen interactions and mechanisms underlying bacterial internalization remain incompletely understood. In this study, we employed an *in vitro* intracellular infection model using bone marrow mesenchymal stem cells alongside an *in vivo* murine osteomyelitis model. Integrated multi-omics, structure-guided molecular docking, and mutation analyses revealed that *S. aureus* promotes the physical recruitment of the host adapter Mprip specifically to the Talin-1 D125 residue. This structural binding triggers the subsequent Talin-1 phosphorylation cascade, which exerts a dual pathological effect: facilitating extensive bacterial internalization and suppressing osteogenic recovery via the PI3K/AKT pathway. Genetic deletion of Mprip or Talin-1 markedly reduced the bacterial burden and restored osteogenesis. Furthermore, through structure-based virtual screening targeting the binding interface at residue D125, we identified a specific small molecule, UM-164. UM-164 effectively disrupted the Mprip/Talin-1 interaction and blocked subsequent Talin-1 phosphorylation. By effectively inhibiting bacterial internalization, UM-164 restricted bacteria to the extracellular space, thereby synergizing with systemic gentamicin to reduce the overall bacterial burden and mitigate bone destruction *in vivo*. In conclusion, the structural binding of Mprip to Talin-1 and the resulting phosphorylation cascade drive *S. aureus* osteomyelitis through a dual mechanism of bacterial internalization and PI3K/AKT-mediated osteogenic suppression. UM-164, a small molecule specifically screened to target the Mprip/Talin-1 D125 interface, presents a potent host-directed strategy to prevent bacterial internalization and potentiate antibiotic efficacy in recalcitrant bone infections.

## Graphical Abstract

**Figure:**
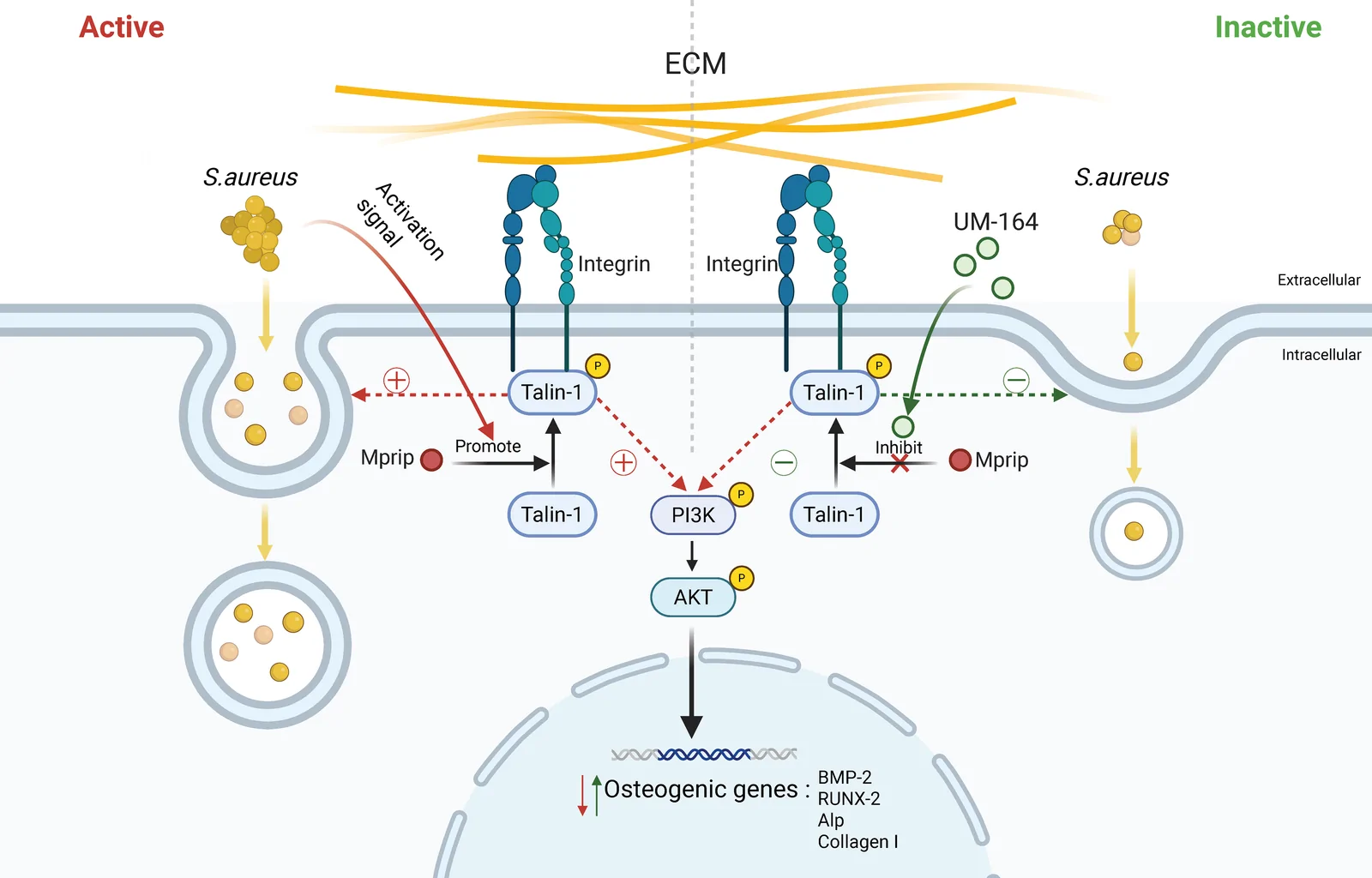

## 1 Introduction

Osteomyelitis is recognized as a severe infectious disease affecting the deep tissues of bone, characterized by prolonged clinical courses and a high incidence of complications. Surgical failure rates exceed 30%, and recurrence is observed in more than 20% of cases (Li et al., 2025; Null, 2025). The global incidence is estimated at 1 to 13 per 100,000 individuals (Jia et al., 2023; Reid and McClung, 2024),with approximately 40% of chronic and recurrent cases experiencing treatment failure (Masters et al., 2022). *Staphylococcus aureus* (*S. aureus*) is identified as the causative pathogen in over 70% of cases, owing to its capacity for tissue invasion, bone colonization, and osteolysis promotion (Zhu et al.; Deng et al., 2023; Saavedra et al., 2024; Santos et al., 2025). The major cause of treatment failure is the ability of *S. aureus* to invade and survive within osteoblasts and mesenchymal cells, forming intracellular reservoirs that markedly reduce the bactericidal efficacy of conventional antibiotics and contribute to infection persistence (Fanous et al., 2025).

Persistent *S. aureus* infection within bone tissue is driven by multiple factors, including the formation of abscess communities, the establishment of glycocalyx on implanted materials, and colonization of the osteocyte-lacunar-canalicular network leading to intracellular infection (Zang et al.; Benoit et al., 2024; Zheng et al., 2024). Recent clinical evidence indicates that the presence of intracellular bacteria significantly increases the risk of treatment failure in *S. aureus* osteomyelitis (Kalinka et al., 2014). Intracellular bacteria not only continuously suppress osteogenic differentiation of osteoblasts and bone marrow mesenchymal stem cells but also facilitate the dissemination and recurrence of infection, acting as a persistent reservoir for chronic osteomyelitis (Tuchscherr et al., 2016). While current research largely focuses on developing advanced drug delivery systems, the pathophysiological mechanisms underlying antibiotic failure against intracellular *S. aureus* remain poorly understood (Voisin et al., 2023; Gao et al., 2024; Admella et al., 2025).

Here, we report that Talin-1-a key integrin-binding protein that regulates cellular adhesion and endocytosis-plays an essential role in bacterial internalization (Hynes et al., 2022; Guo et al., 2023; Wang et al., 2024; Cheville et al., 2025). Mechanistically, we identified that *S. aureus* hijacks this process by driving the physical recruitment of the host adapter Mprip specifically to the Talin-1 D125 residue. This structural interaction serves as a critical prerequisite for the subsequent phosphorylation cascade of Talin-1, thereby accelerating bacterial entry and suppressing osteogenic recovery via the PI3K/AKT pathway. Notably, genetic deletion of Mprip/Talin-1 or pharmacological blockade of their D125 binding interface enhances antibiotic bactericidal efficiency and alleviates bacterial burden and bone destruction in *S. aureus*-induced osteomyelitis. These findings elucidate a critical mechanism of *S. aureus* internalization during osteomyelitis progression and establish a foundation for developing host-targeted strategies that potentiate antibiotic efficacy in bone infections.

## 2 Materials and methods

### 2.1 Bacterial cultivation

The Staphylococcus aureus strain ATCC 25923 was utilized as the infectious agent in this study. This strain was specifically selected as the primary experimental model because it is a globally recognized “gold standard” for investigating staphylococcal pathogenesis, particularly in the context of bone and joint infections. Unlike uncharacterized clinical isolates, ATCC 25923 possesses a well-characterized genomic profile and consistent expression of key virulence factors, such as fibronectin-binding proteins, which are essential for mediating bacterial internalization into host cells. The use of this standardized reference strain ensures high experimental reproducibility and provides a reliable baseline for mapping the Mprip/Talin-1 signaling axis. For experimental procedures, bacteria were cultured in Tryptic Soy Broth (TSB) at 37 °C with shaking at 200 rpm until the mid-logarithmic growth phase was reached to ensure optimal metabolic activity and invasive capacity.

### 2.2 Osteomyelitis mouse model

All animal experiments were conducted in accordance with the guidelines approved by the Institutional Animal Care and Use Committee (IACUC) of the Shanghai Institute of Immunology and Infection, Chinese Academy of Sciences (Approval No. A2025018). Adult male C57BL mice (8 weeks old) were obtained from Shanghai Jiesijie Laboratory Animal Co., Ltd. and acclimatized for 3 days prior to surgery. To establish the osteomyelitis model, mice were anesthetized with isoflurane, and the surgical site was shaved and disinfected with 75% ethanol. Following a medial parapatellar incision to expose the distal femur, a transcortical channel was created into the medullary cavity using a 26-gauge needle. Subsequently, 2 μL of PBS (control) or PBS containing 10^^6^ CFU of *S. aureus* was locally injected into the marrow cavity using a microsyringe. Crucially, the injection site was immediately sealed with sterile bone wax to prevent bacterial leakage into the surrounding soft tissues. The incision was then sutured, and mice were closely monitored until full recovery from anesthesia. Following the establishment of this local infection, therapeutic interventions were administered systemically to evaluate drug efficacy. To ensure dosing precision, all therapeutic agents were prepared in sterile PBS, and administration volumes were strictly adjusted to 10 μL/g of body weight. The control and bacterial infection groups received daily intraperitoneal (i.p.) injections of PBS as a vehicle control. The antibiotic treatment group was administered gentamicin at a dosage of 20 mg/kg body weight daily via i.p. injection, ensuring systemic bioavailability. For the combined therapy group, a mixture of gentamicin (20 mg/kg) and the small-molecule inhibitor UM-164 (8 mg/kg) was administered daily via i.p. injection. Body weights were monitored every two days throughout the 30-day treatment period to maintain accurate weight-based dosing. All systemic treatments were maintained for a duration of one month, after which (on postoperative day 30), the mice were euthanized, and femoral tissues were harvested for Micro-Computed Tomography (Micro-CT) analysis, histological evaluation, and immunofluorescence staining (n = 6 per group).

### 2.3 Cell culture

Cells were aseptically collected from the femurs and tibias of 8-week-old male C57BL mice. The marrow cavities were flushed with culture medium to obtain a single-cell suspension, which was then seeded into culture dishes. The cells were incubated at 37 °C in an atmosphere containing 5% CO_2_ for 48 h. Non-adherent cells were removed, and fresh medium was added every 48 hours until the adherent cells reached confluence. Second-passage (P2) bone marrow mesenchymal stem cells (BMSCs) were used for subsequent experiments.

### 2.4 Co-culture model of bacteria and BMSCs

Primary BMSCs at passage 2 (P2) were seeded into 6-well plates and cultured in α-MEM supplemented with 10% fetal bovine serum. Prior to bacterial inoculation, the culture medium was replaced with antibiotic-free α-MEM. The cells were then infected with *S. aureus* at a multiplicity of infection (MOI) of 200 (defined as the ratio of bacterial cells to host cells) for 2 hours. Following this initial infection period, the cells were thoroughly washed with PBS. To specifically eliminate extracellular bacteria and establish a true intracellular infection model, the cells were subsequently cultured in a fresh medium containing lysostaphin (10 μg/mL) and gentamicin (40 μg/mL). The infected BMSCs were then harvested and processed for subsequent analyses.

### 2.5 Live/dead staining assay

Wash the cells thoroughly with PBS 2–3 times to remove any residual esterase activity. After removing the PBS, the adherent cells were treated with a sufficient volume of calcein-acetoxymethyl ester-propidium iodide staining solution (PF00007, Proteintech, China) (prepared according to the instructions). The cells were then wrapped in tin foil and incubated at room temperature in the dark for 20 minutes. The stained cells were then observed using a fluorescence microscope.

### 2.6 Alkaline phosphatase staining

Cells were visualized by alkaline phosphatase activity using the BCIP/NBT chromogenic substrate kit (C3206, Beyotime Biotechnology, China) according to the provided protocol. Subsequent to a PBS wash step, fixation was carried out for 15 min using 4% paraformaldehyde maintained at 4 °C. Incubation with the BCIP/NBT solution, freshly mixed following the manufacturer’s specifications, proceeded for 30 min under conditions of darkness. Termination of the chromogenic reaction was achieved by removal of the substrate solution and thorough washing with PBS.

### 2.7 RT-qPCR

To isolate high-quality total RNA, a dedicated RNA purification protocol was rigorously followed. Cultured cells were first lysed utilizing a specialized reagent designed to immediately stabilize and protect RNA integrity. The resultant lysate was processed according to the optimized workflow of a commercial RNA isolation system, which effectively separates RNA from DNA, proteins, and other cellular contaminants through a series of selective binding and washing steps on a silica-based membrane column. The purified RNA was subsequently eluted in nuclease-free water. The concentration and purity of the eluted RNA were precisely assessed using a microvolume spectrophotometer (NanoDrop™ One, Thermo Fisher Scientific, MA, USA) to ensure suitability for downstream applications. After quantification, reverse transcription (cDNA). The quantitative real-time polymerase chain reaction (qRT-PCR) was subsequently performed on a thermal cycler platform with a SYBR Green-based master mix to fluorescently monitor amplification kinetics in real-time. All reactions were carried out in technical triplicates with appropriate negative controls. The oligonucleotide primer pairs employed for the amplification of each target gene are comprehensively listed in **Additional File 1: Supplementary Table 1**.

### 2.8 Western blot

The culture medium in the 6-well plate was washed, and protease and phosphatase inhibitors were pre-added at a concentration of 1% in radio immunoprecipitation assay buffer (P0013B, Beyotime, China) and then added to the plate to lyse the cells. The cell lysates were then thoroughly disrupted by sonication and the protein concentration was determined using the bicinchoninic acid assay to ensure consistent protein loading. After boiling the protein at 95°C for 10 minutes, add it to the lanes and add 10–20 uL to each well. After electrophoresis, transfer the colloid to a PVDF membrane activated with methanol. The membrane was then blocked with 5% skim milk and washed three times with Tris-buffered Saline with Tween-20 (TBST) for 10 minutes. The membrane was then incubated with the target antibody overnight at 4 °C. A secondary antibody matched to the primary antibody was added and incubated on a shaker for 1 hour. Finally, develop with electrochemiluminescence developer. Protein bands were finally detected using a chemiluminescence imaging system (Touch Imager, e-BLOT, China). Antibodies used: BMP-2 (A0231, ABclonal, China), Runx-2 (A11753, ABclonal, China), Alp (A0514, ABclonal, China), Collagen-1 (A21059, ABclonal, China), Talin-1 (14168-1-AP, Proteintech, China), FAK (66258-1-Ig, Proteintech, China), Pan phospho-Serine/Threonine Mouse mAb (AP1067, ABclonal, China), Pan phospho-Tyrosine Mouse mAb (AP0973, ABclonal, China), Mprip (D8G8R, Cell Signaling Technology, USA). Protein marker: PL00003 (10–310 kDa, Proteintech, China).

### 2.9 Immunofluorescence

Following PBS washing to remove residual medium, infected cell membranes were permeabilized by treatment with 0.3% Triton X-100 in PBS. Cells were incubated in blocking solution for 1 hour to prevent non-specific binding. Primary antibody incubation was performed overnight at 4 °C using mouse monoclonal anti-Talin-1 (clone YQ-16, Santa Cruz Biotechnology) and rabbit polyclonal anti-Mprip (20040-1-AP, Proteintech), diluted in antibody dilution buffer. The following day, cells were thoroughly rinsed three times with TBST before application of species-matched secondary antibodies conjugated to fluorophores for 1 hour at room temperature protected from light. Unbound secondary antibodies were removed by additional TBST washes, and nuclear counterstaining was accomplished with 4’,6-diamidino-2-phenylindole (DAPI). Coverslips were mounted using antifade mounting medium, and images were captured using a fluorescence microscope (Leica DMi8, Leica Microsystems).

### 2.10 Intracellular bacterial quantification

To strictly differentiate intracellular from extracellular bacteria, an antibiotic protection assay was performed. Following infection, the BMSCs were washed three times with PBS to remove non-adherent bacteria. The cells were then incubated in a medium containing lysostaphin (10 μg/mL) and gentamicin (40 μg/mL) for 1 hour at 37 °C. Lysostaphin specifically degrades the cell wall of extracellular S. aureus, while gentamicin (which has poor membrane permeability) eliminates any remaining surface-bound bacteria. After enzymatic and antibiotic treatment, the cells were washed again and lysed with 0.3% Triton X-100 to release the internalized bacteria. The lysates were then serially diluted and plated onto agar plates for CFU counting.

### 2.11 Cell transfection

SiRNAs targeting Talin-1, Mprip, His and Myc were purchased from General Biol Co., Ltd. (China). For mutation assays, the Talin-1 mutant plasmids were commercially synthesized by General Biol Co., Ltd. (China) with sequence fidelity confirmed by Sanger sequencing. Cells were seeded in 6-well plates and transfected using Lipofectamine™ RNAiMAX (Thermo Fisher Scientific, USA) at approximately 80% confluency. First, add SiRNA to Opti-MEM Reduced Serum Medium (Gibco, Thermo Fisher Scientific, USA) without shaking. Then add transfection reagent and mix for 15 minutes. Discard the serum-containing medium and replace it with cells. Knockdown efficiency was assessed by western blotting. Primer sequences in **Additional File 1: Supplementary Table 2**.

### 2.12 RNA‐sequencing

To evaluate transcriptomic alterations, infected and control BMSCs (n = 3 independent biological replicates per group) were trypsinized and harvested. Total RNA was extracted using TRIzol reagent (Invitrogen), and RNA integrity and quantity were assessed using an Agilent 2100 Bioanalyzer as part of the rigorous quality control (QC) metrics. For each sample, 3 μg of high-quality RNA was used for library preparation. RNA libraries were constructed with unique index codes according to the manufacturer’s instructions, and fragments of 250–300 bp were purified using the Agencourt AMPure XP system. Sequencing was performed on the Illumina platform by MetWare Biotechnology Co., Ltd. (Wuhan, China). For bioinformatics analysis, differential expression was evaluated using the Benjamini-Hochberg method to control the false discovery rate. Genes with a corrected *P* value ≤ 0.05 and a |log_2_ fold change| ≥ 0.5 were defined as significantly differentially expressed.

### 2.13 CCK‐8

Cells were counted using a CCK-8 kit (Sigma-Aldrich, St. Louis, MO, USA) and seeded at 5,000 cells/well in 96-well plates for 24–48 hours. Reagents were then added and incubated in the dark. OD values were measured at 450 nm using a microplate reader. The experimental design included treatment groups with concentrations of 0, 0.001, 0.01, 0.1, 1, and 10 μmol/L.

### 2.14 Micro‐CT analysis

We employed a SkyScan 1276 Micro-CT system for scanning and subsequent three-dimensional (3D) reconstruction. Image analysis focused on a region of interest spanning slices 10 to 110, using the growth plate slice (designated as 0 mm) as the reference point. Grayscale contrast was set within the range of 68 to 255. Using CTAn software (Bruker Micro CT), we performed 3D analysis, bone mineral density (BMD) assessment, and generated 3D models, which were subsequently modified in CT Vox software (Bruker Micro CT).

### 2.15 Histological analysis

Femoral specimens were harvested from the mice and carefully denuded of surrounding soft tissues, such as muscle and fascia. The specimens were then fixed in 4% paraformaldehyde for 48 hours at 4 °C. To ensure the preservation of protein epitopes and cellular architecture for subsequent immunohistochemical analysis, the fixed bones were decalcified in 10% neutral EDTA (pH 7.4) at room temperature for approximately 8 weeks, with the decalcification solution refreshed every 3 days. This prolonged but gentle chelating process was specifically chosen to avoid the harsh acidic environment that typically degrades sensitive epitopes. Following complete decalcification (confirmed by the needle-puncture test), the specimens were dehydrated through a graded series of ethanol, embedded in paraffin, and cut into 5-μm-thick longitudinal sections. These sections were further processed for immunofluorescence staining to evaluate the spatial distribution and expression levels of target proteins within the bone tissue.

### 2.16 Mass spectrometry analyses

For mass spectrometry (MS) analysis, BMSCs cultured in two 10-cm dishes were harvested to generate whole-cell lysates. Immunoprecipitation of the lysates was carried out using an anti-Talin-1 antibody (Beyotime, China; Cat. no. P2179S). The co-immunoprecipitated (Co-IP) complexes were extensively washed with PBS while bound to the beads, and MS analysis was subsequently performed directly on the bead-bound complexes. Chromatographic separation was performed using a Vanquish Neo UHPLC system (Thermo Fisher Scientific). Following nano-flow liquid chromatography, peptides were analyzed by data-independent acquisition on an Orbitrap Astral high-resolution mass spectrometer (Thermo Scientific).

### 2.17 Protein purification

The target protein was purified using nickel-NTA agarose beads. After incubating the cell lysate with the resin in binding buffer (20 mM imidazole), the beads were subjected to a series of washes using a wash buffer containing 20 mM imidazole to remove weakly associated and non-specifically bound proteins. Target proteins were subsequently eluted from the beads using an elution buffer with a high concentration of imidazole (250 mM). The final elution fraction was analyzed by sodium dodecyl sulfate-polyacrylamide gel electrophoresis.

### 2.18 Molecular docking and surface plasmon resonance analysis

Virtual Screening: To identify potential small-molecule inhibitors targeting the Mprip/Talin-1 interface, a structured virtual screening workflow was implemented. The structure of the Talin-1 head domain was obtained using AlphaFold3 and equilibrated via energy minimization. A library of approximately 70,000 compounds was screened using Glide (Schrödinger LLC) in standard-precision (SP) docking mode. Candidates were ranked by docking scores and further refined using the MM/GBSA (Molecular Mechanics/Generalized Born Surface Area) method to estimate binding free energies. The D125 pocket was defined as the target site based on preliminary mutational data. UM-164 was selected as the lead candidate due to its superior binding affinity, favorable hydrophobic interactions within the D125 pocket, and low false-positive potential in benchmarking.

SPR Analysis: The direct binding kinetics between UM-164 and Talin-1 were characterized using a Biacore X100 system (GE Healthcare). Purified recombinant Talin-1 protein was covalently immobilized onto a CM5 sensor chip via standard amine coupling to a target level of 5,000 resonance units (RU). A flow cell without protein served as a reference to account for non-specific binding. UM-164 was prepared in running buffer (PBS-T with 5% DMSO) and injected at concentrations ranging from 0.3125 to 10 μM at a flow rate of 30 μL/min. Double-reference subtraction (subtracting both reference cell signal and buffer injections) was performed to eliminate systemic artifacts and solvent effects. The resulting sensorgrams were globally fitted to a 1:1 Langmuir binding model using Biacore Evaluation Software to derive the association rate (ka), dissociation rate (kd), and equilibrium dissociation constant (KD). Experiments were performed in triplicate to ensure reproducibility and consistency.

### 2.19 Statistical analysis

All quantitative data are expressed as mean ± standard deviation (SD), with the number of biological or technical replicates (n = 3 or 6) indicated for each experimental group. Statistical significance was defined as *p* < 0.05. For comparisons between two groups, a two-tailed unpaired Student’s *t*-test was applied. For comparisons among three or more groups, one-way analysis of variance (ANOVA) was used, followed by appropriate *post hoc* tests. All statistical analyses were conducted using GraphPad Prism (version 8.0, GraphPad Software, Boston, MA, USA).

## 3 Results

### 3.1 Effects of intracellular bacteria on BMSCs osteogenesis

**Figure 1:**
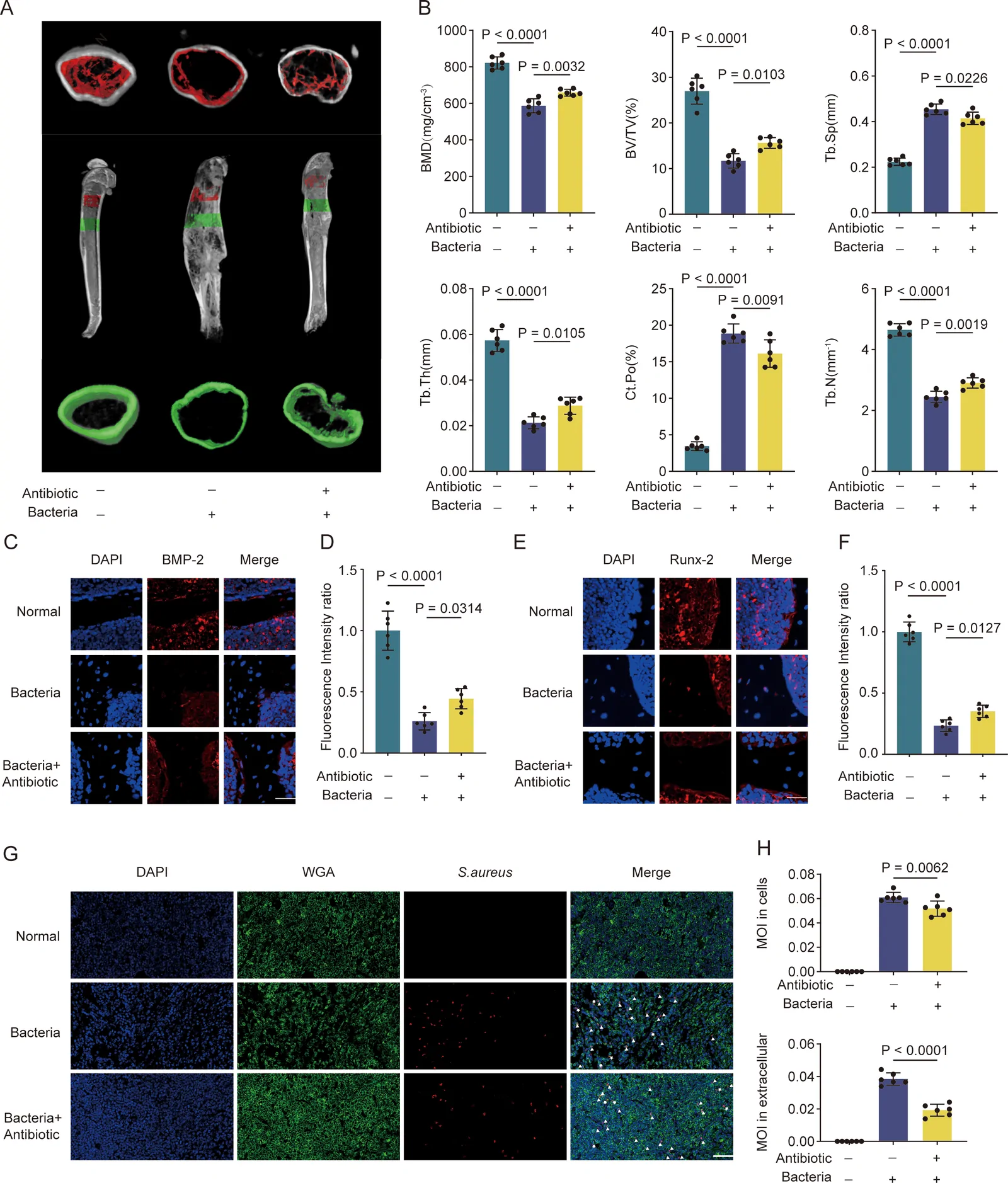
Intracellular bacteria impair BMSCs osteogenesis despite antibiotic treatment. **(A)** Micro-CT analysis showing 3D overview and 2D cross-sectional views of femoral bones from the indicated experimental groups at 30 days post-intervention. **(B)** Bone-related parameters, including bone mineral density (BMD), bone volume/total volume (BV/TV), trabecular separation (Tb.Sp), trabecular thickness (Tb.Th), cortical porosity (Ct.Po), and trabecular number (Tb.N), were quantified (n = 6). **(C, D)** Immunofluorescence staining of BMP-2 in the femoral bone tissues of mice, with subsequent quantification (original magnification ×63; scale bar = 50 µm). **(E, F)** Immunofluorescence staining of Runx-2 in the femoral bones, along with quantitative analysis. **(G)** Histological analysis of bone tissue sections stained for bacteria in the control, bacterial, and antibiotic-treated groups. Cell nuclei are stained blue with DAPI, cell membranes are stained green with wheat germ agglutinin, and *S. aureus* is stained red. In the merged image, triangles indicate intracellular bacteria, while pentagrams denote extracellular bacteria. **(H)** Quantitative assessment of intracellular and extracellular bacteria and their corresponding MOI.

To investigate the impact of intracellular bacteria on osteogenesis of BMSCs, we first established a murine osteomyelitis model with a 2-week infection duration. The groups included antibiotic treatment, bacterial infection, and bacterial infection combined with antibiotics. Micro-CT analysis was conducted on the cortical and cancellous bone of mouse femurs (**Figure 1A**), accompanied by quantitative assessment of bone-related parameters (**Figure 1B**). Results demonstrated a significant reduction in bone quality in bacterial-infected mice, with some partial recovery following adequate antibiotic treatment; however, the overall therapeutic efficacy remained suboptimal. Subsequently, immunofluorescent staining of tissue sections revealed markedly decreased expression of BMP-2 and Runx-2, which showed partial restoration after antibiotic therapy (**Figures 1C–F**). We then performed *S. aureus* staining on the mouse femurs, and the results showed (**Figures 1G, H**) that after antibiotic treatment, the number of extracellular bacteria was greatly reduced, but there were still residual bacteria in the cells. The above results show that the presence of bacteria in the embryo may be the main cause of persistent bone loss in osteomyelitis. In conclusion, both *in vivo* and *in vitro* experiments demonstrate that bacterial infection exerts a sustained inhibitory effect on BMSCs osteogenesis, even when treated with antibiotic.

### 3.2 RNA‐Sequencing reveals altered phosphorylation and upregulated adhesion pathway

**Figure 2:**
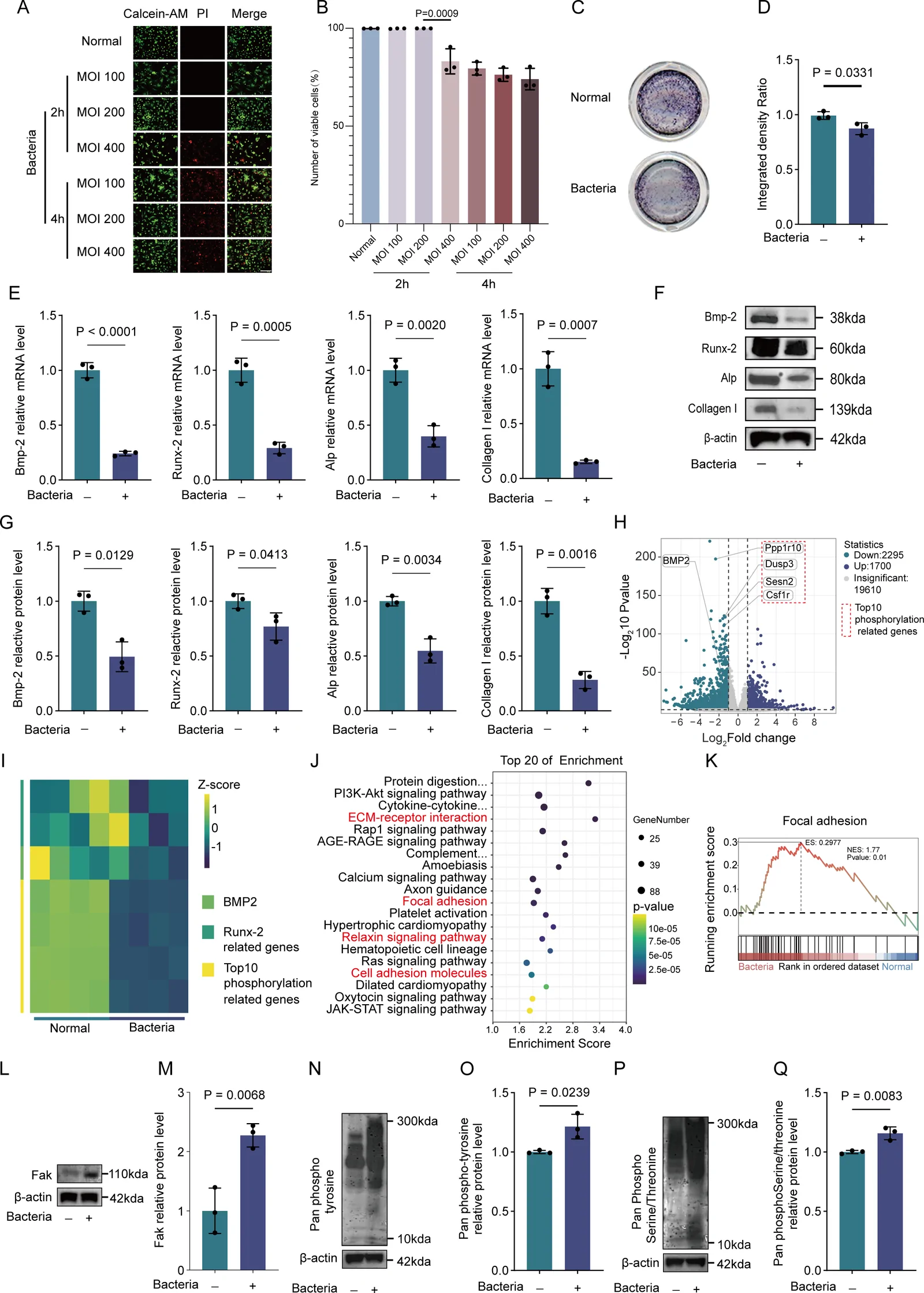
Transcriptome sequencing demonstrated increased global phosphorylation. **(A, B)** Live/dead cell staining and quantification following bacterial infection at different MOIs (100, 200, 400) and time points (2 h, 4 h) (scale bar = 50 µm; *n* = 3). **(C, D)** ALP staining and fluorescence intensity quantification before and after bacterial infection. **(E)** RT-qPCR analysis of BMP-2, Runx-2, Alp, and Collagen I expression in control and bacteria groups. **(F, G)** Western blotting and corresponding quantitative analysis of BMP-2, Runx-2, Alp and Collagen I in control and bacteria groups. **(H)** Volcano plot of transcriptome sequencing differential expression between the control group and bacteria-infected group [Dashed box: phosphorylation-related genes (Top10)]. **(I)** Heatmap of differential gene expression. **(J)** Top 20 keggenriched gene pathways based on transcriptome sequencing between the control group and bacteria-infected group (Red font: Extracellular matrix and adhesion-related pathways). **(K)** GSEA plot of the focal adhesion signaling pathway. **(L, M)** Western blot analysis and quantitative measurement of Fak protein expression in the control and bacteria-infected groups (n = 3). **(N, O)** Western blot analysis and quantitative measurement of global phosphotyrosine levels in the control and bacteria-infected groups. **(P, Q)** Western blot analysis and quantitative measurement of global phosphoserine/threonine levels in the control and bacteria-infected groups.

To further explore the mechanism by which bacteria influence osteogenesis, we innovatively developed an *in vitro* model. BMSCs were co-cultured with *S. aureus* at an MOI of 200 for 2 hours in antibiotic-free medium (**Figures 2A, B**), followed by the application of lysozyme and gentamicin to eliminate extracellular bacteria. After 24 hours, the osteogenic markers in BMSCs were assessed. Alp staining further confirmed decreased alkaline phosphatase activity in infected BMSCs (**Figures 2C, D**). Additionally, RT-qPCR analysis showed downregulation of osteogenic genes BMP-2, Runx-2, Alp, and Collagen I (**Figure 2E**), indicating bacterial suppression of osteogenic gene expression. Western blot analysis of BMP-2, Runx-2, Alp, and Collagen I corroborated the findings, demonstrating a significant decrease in the protein levels of osteogenic markers following bacterial infection (**Figures 2F, G**). To further elucidate the mechanisms by which bacteria influence BMSCs, we conducted transcriptomic sequencing of the samples (**Additional File: Supplementary Figure 1**). Differential gene expression was explored through volcano plots, heatmaps, kegg pathway analysis, and gene set enrichment analysis (GSEA). Notably, aside from a decrease in osteogenic marker expression, four of the top ten most significantly differentially expressed genes were associated with phosphorylation processes, namely Ppp1r10, Dusp3, Sesn2, and Csf1r-all of which were downregulated (**Figures 2H, I**). Focusing on phosphorylation-related pathways, enrichment analysis identified several cell adhesion pathways among the top twenty, including ECM-receptor interaction, relaxin signaling pathway, cell adhesion molecules, and focal adhesion (**Figure 2J**). Within these, the overexpression of focal adhesion components suggests that bacterial impact on osteogenic differentiation may involve modulation of cell adhesion processes (**Figure 2K**). To investigate this hypothesis, we examined the expression of FAK, a kinase in adhesion signaling. Results demonstrated that bacterial exposure significantly increased FAK expression (**Figures 2L, M**). Furthermore, we validated elevated levels of global tyrosine phosphorylation (**Figures 2N, O**) and serine/threonine phosphorylation (**Figures 2P–O**), consistent with enhanced phosphorylation signaling in the bacterial group. In summary, bacteria may promote their internalization into BMSCs by upregulating phosphorylation of adhesion-related proteins, thereby enhancing cell adhesion and subsequently impacting osteogenic differentiation.

### 3.3 Identified talin-1 hyperphosphorylation in adhesion pathway *via* proteomics/phosphoproteomics co-analysis

**Figure 3:**
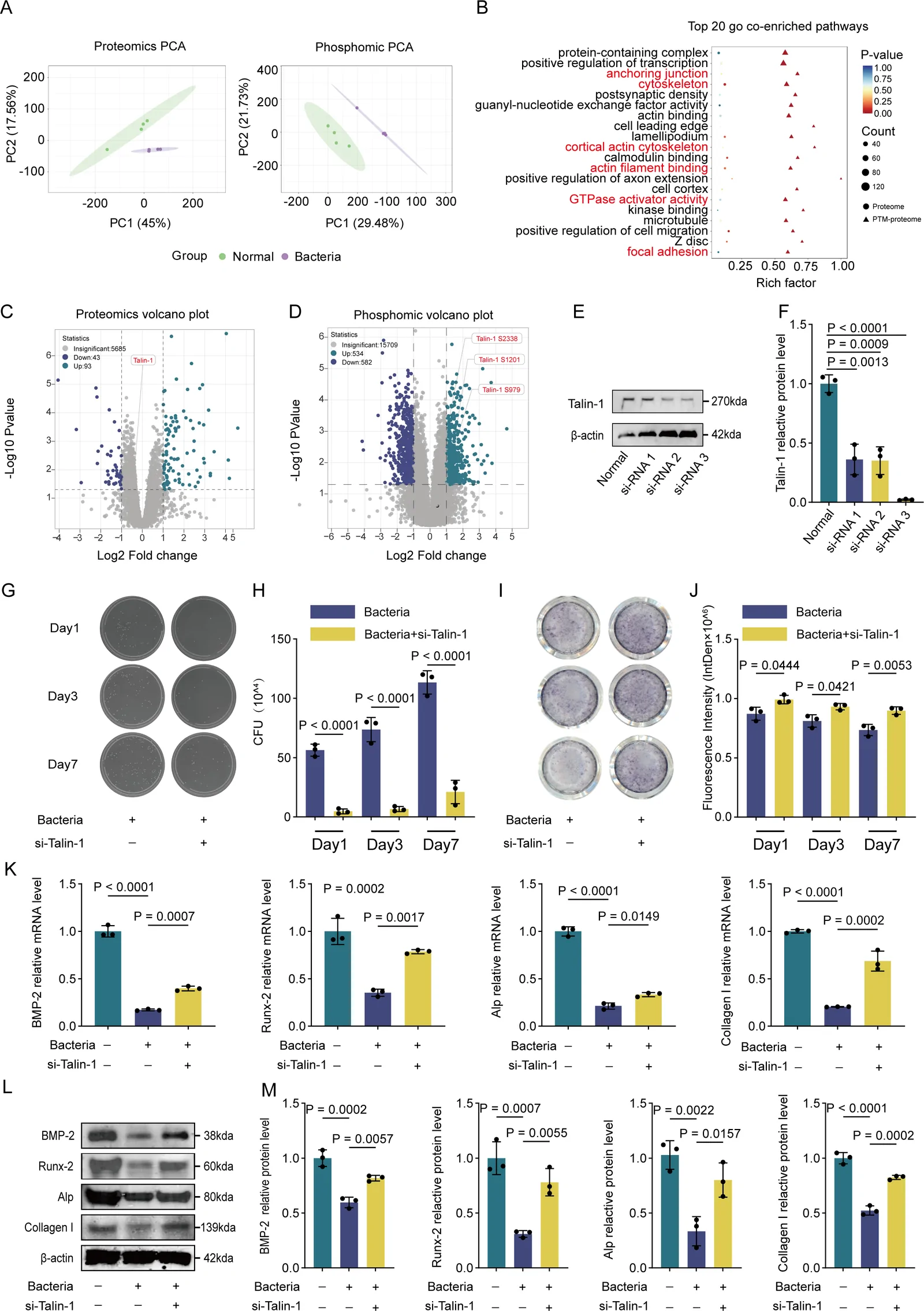
Integrated omics reveals talin-1 as key for bacterial internalization. **(A)** PCA analysis of proteomics and phosphoproteomics. **(B)** Top 20 significantly enriched go pathways shared (Red font: extracellular matrix and adhesion-related pathways). **(C)** Volcano plot of proteomics data (No significant difference in Talin-1 protein expression). **(D)** Volcano plot of phosphoproteomics data (all significantly altered Talin-1 modification sites are up-regulated). **(E, F)** Western blotting and quantitative analysis of Talin-1 transfection experiments. **(G, H)** Comparison of intracellular colony formation at 1/3/7 days: si-Talin vs. Bacterial groups (n = 3). **(I, J)** ALP fluorescence staining and quantitative analysis of fluorescence intensity at 1/3/7 days between si-Talin and bacterial groups. **(K)** RT-qPCR analysis of BMP-2, Runx-2, Alp, and Collagen I expression in control, bacteria and si-Talin-1 groups. **(L, M)** Western blotting and corresponding quantitative analysis of BMP-2, Runx-2, Alp and Collagen I in control, bacteria, and si-Talin-1 groups.

To further elucidate the mechanism by which bacteria influence osteogenesis of BMSCs through phosphorylation-mediated adhesion pathways, we conducted an integrated proteomics and phosphoproteomics analysis to explore the pathogenic mechanisms at a molecular level. PCA revealed significant differences between groups (**Figure 3A**). Notably, among the top 20 pathways exhibiting significant phosphorylation alterations (**Figure 3B**), numerous pathways related to cell adhesion were identified, including anchoring junctions, cytoskeleton organization, cortical actin cytoskeleton, actin filament binding, GTPase activator activity, and focal adhesion. Focusing on focal adhesion, protein phosphorylation level analysis highlighted a prominent differential, especially in Talin-1, which showed marked phosphorylation changes despite relatively modest differences at the total protein level (**Figures 3C, D**). This suggests that phosphorylation of proteins such as Talin-1 might be critical in regulating the adhesion pathway, thereby affecting osteogenic differentiation of BMSCs. Based on this, Talin-1 was designated as a focal point for subsequent functional validation. We first employed gene knockdown *via* transfection reagents to suppress Talin-1 expression (**Figures 3E, F**). To ensure that Talin-1 knockdown and subsequent bacterial challenge did not impair cellular viability, a CCK-8 assay was performed, which demonstrated that BMSC proliferation and cell viability remained uncompromised across the 1, 3, and 7days experimental time course (**Additional File: Supplementary Figure 3**). Functional assays demonstrated that, compared with controls, suppression of Talin-1 significantly reduced intracellular bacterial load after co-cultivation with bacteria for 2 hours, followed by culture in gentamicin and lysozyme-containing media for 1, 3, and 7 days, with subsequent cell lysis, dilution, and colony counting (**Figures 3G, H**). Furthermore, ALP staining was conducted at 1, 3, and 7 days post-infection to compare osteogenic activity between si-Talin-1 knockdown and control groups (**Figures 3I, J**).This indicates that phosphorylated Talin-1 plays a pivotal role in bacterial internalization, and its inhibition impairs bacterial invasion, effectively restricting bacteria to the extracellular space and enhancing antibiotic efficacy. Further, we assessed the impact of Talin-1 phosphorylation inhibition on osteogenic gene expression (**Figure 3K**) and validated osteogenic markers BMP-2, Runx-2, Alp, and Collagen I by western blot and quantitative analysis (**Figures 3L, M**). The results demonstrated that phosphorylation suppression of Talin-1 restored osteogenic marker expression, indicating a rescue of osteogenic potential.

### 3.4 Mprip interacts with talin-1 and regulates Talin-1 phosphorylation and osteogenic differentiation in BMSCs

**Figure 4:**
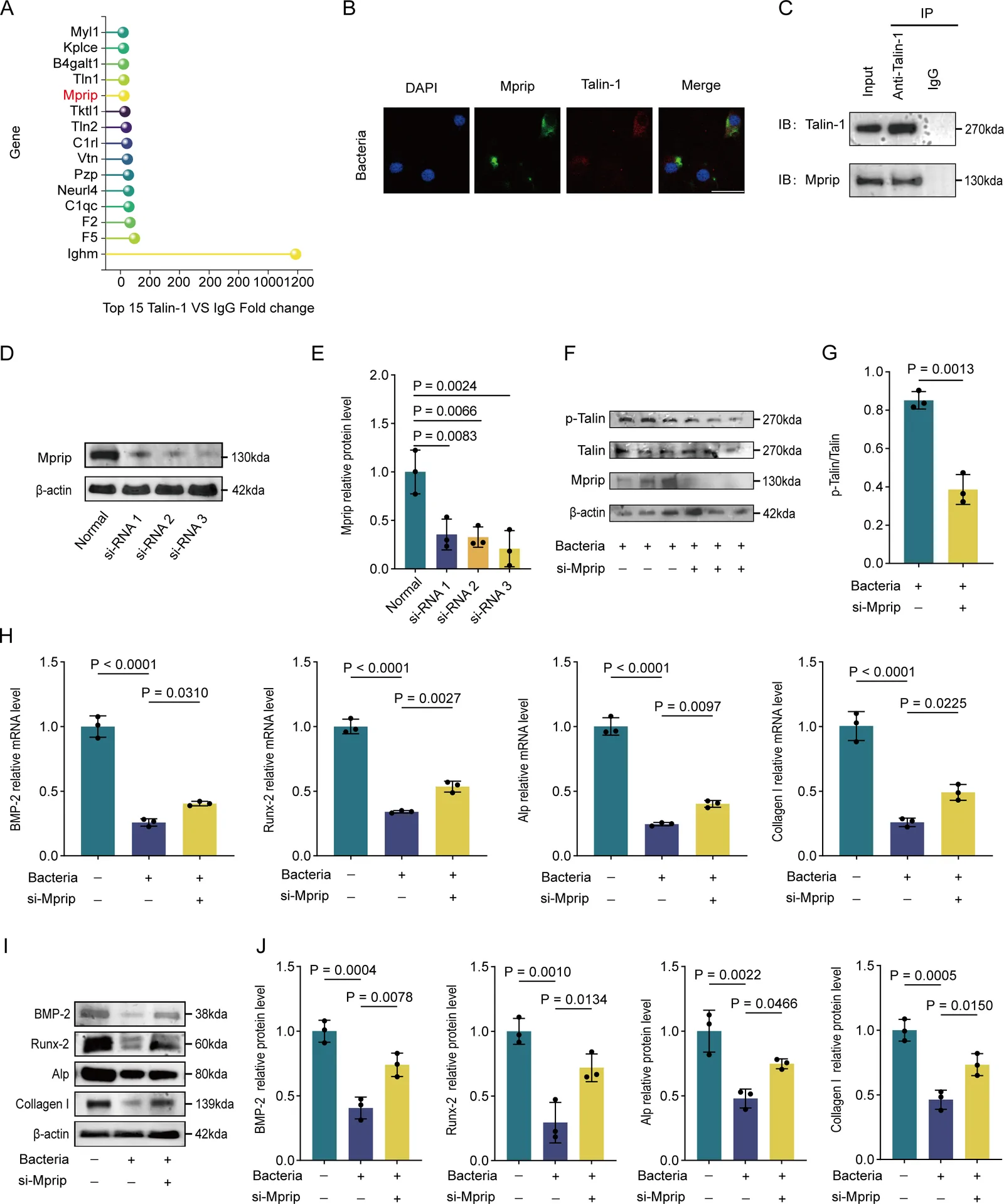
Mprip physically interacts with Talin-1 to trigger its subsequent phosphorylation. **(A)** Ranked list of Talin-1 interacting proteins. **(B)** Confocal fluorescence images showing colocalization of host Mprip and Talin-1 upon *S. aureus* infection. Blue indicates DAPI; green, Mprip protein; red, Talin-1 protein. (scale bar = 50 µm). **(C)** Co-IP assay demonstrating the interaction between Talin and Mprip. **(D)** Transfection efficiency of siRNA targeting Mprip (si-Mprip). **(E)** Quantitative analysis of si-Mprip transfection (n = 3). **(F, G)** Western blot analysis showing that knockdown of Mprip decreases the protein expression level of phosphorylated Talin. **(H)** RT-qPCR analysis of osteogenic markers BMP-2, Runx-2, Alp, and Collagen I in control, bacterial, and si-Mprip groups. **(I, J)** Western blot analysis and quantification of BMP-2, Runx-2, Alp, and Collagen I expression in control, bacterial, and si-Mprip groups (n = 3).

Upon preliminary identification of the bacterial pathogenic protein, we sought to elucidate its upstream and downstream signaling pathways. First, Talin-1 and its interacting proteins were isolated from cellular lysates using magnetic bead-based immunoprecipitation, followed by mass spectrometry analysis. The resulting proteomic data, ranked by fold change comparing Talin-1 versus IgG control, are shown in (**Figure 4A**). Subsequently, we performed immunofluorescence staining for Mprip and Talin-1. Fluorescence microscopy revealed significant spatial overlap between the two proteins (**Figure 4B**). Co-IP assays confirmed a direct protein-protein interaction between Talin-1 and Mprip (**Figure 4C**). To validate Mprip’s functional role, we transfected cells with Mprip using Lipofectamine 3000 (**Figures 4D, E**). Silencing of Mprip significantly decreased Talin-1 phosphorylation relative to the control group (**Figures 4F, G**). Next, osteogenic differentiation markers were evaluated in Mprip-transfected cells by RT-qPCR analysis of BMP-2, Runx2, Alp, and Collagen I, showing significant restoration of osteogenic gene expression (**Figure 4H**). Western blotting and quantitative analyses of these osteogenic proteins corroborated the mRNA findings, demonstrating marked recovery post-Mprip transfection (**Figures 4I, J**).

**Figure 5:**
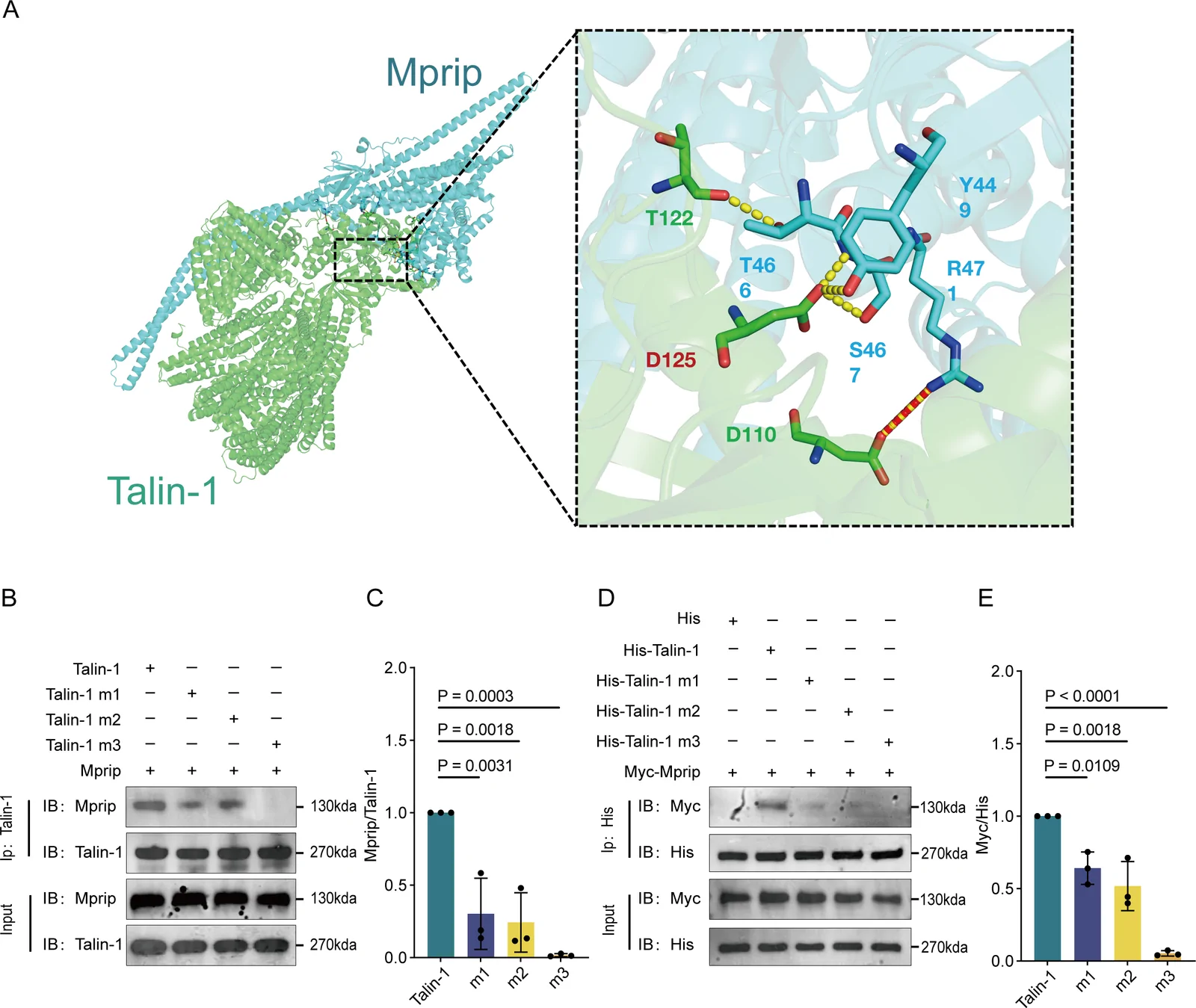
Mprip binds strongest to talin-1 at residue D125. **(A)** Molecular docking simulation of Mprip with Talin-1, highlighting binding sites at [D110 (m1), T122 (m2), and D125 (m3)]. **(B–E)** Co-IP assays and quantitative analysis of Talin-1 mutants (D125, T122, D110) interacting with Mprip (n = 3).

This suggests that S. aureus inhibits osteogenesis by triggering the structural recruitment of Mprip, which subsequently induces the Talin-1 phosphorylation cascade. To further elucidate the molecular mechanism, AlphaFold3-based structural modeling was employed to identify the physical docking interface; the results identified Talin-1 residues D110, T122, and D125 as putative Mprip binding sites (**Figure 5A**). To pinpoint the critical residue required for this interaction, Talin-1 mutants targeting these three sites [D110 (m1), T122 (m2), and D125 (m3)] were generated. Pulldown assays showed a considerably reduced binding affinity between Mprip and the Talin-1 m3 mutant, indicating that residue D125 is the predominant interface for the physical interaction between Mprip and Talin-1 (**Figures 5B–E**). Consequently, this interaction at D125 serves as the essential structural prerequisite for the subsequent phosphorylation of Talin-1 and the activation of downstream signaling.

### 3.5 Talin-1 impairs BMSCs osteogenesis *via* PI3K-AKT activation

**Figure 6:**
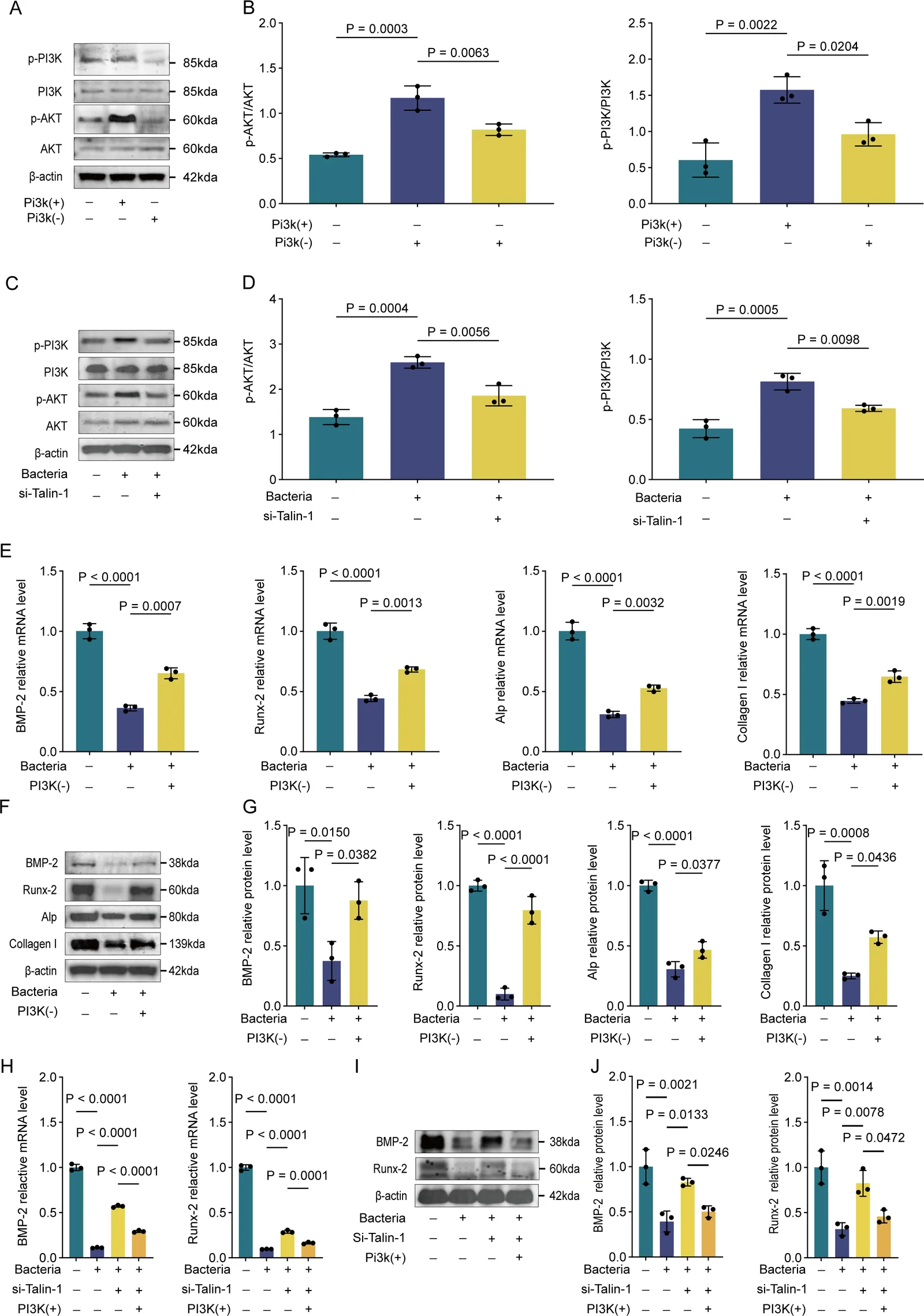
Talin-1 inhibits osteogenesis by activating the PI3K-AKT pathway. **(A, B)** Western blot analysis and quantitative comparison of PI3K–AKT activator (740 Y-P at a concentration of 20 μM) and inhibitor (PI3K/AKT-IN-1 (2.62 µM)) treatments. **(C, D)** Western blot analysis and quantitative measurement of PI3K/p-PI3K and AKT/p-AKT in Control, Bacteria, and Bacteria + si-Talin-1 groups. **(E)** RT-qPCR analysis of BMP-2, Runx-2, Alp, and Collagen I expression in Control, Bacteria, and Bacteria + PI3K (-) groups, PI3K (-): PI3K/AKT pathway inhibitor. **(F, G)** Western blot analysis and quantification of BMP-2, Runx-2, Alp, and Collagen I in Control, Bacteria, and Bacteria + PI3K (-) groups. **(H)** RT-qPCR analysis of BMP-2 and Runx-2 in Control, Bacteria, Bacteria + si-Talin-1, and Bacteria + si-Talin-1 + PI3K(+) groups. **(I, J)** Western blot analysis and quantitative assessment of BMP-2 and Runx-2 in Control, Bacteria, Bacteria + si-Talin-1, and Bacteria + si-Talin-1 + PI3K (+) groups (n = 3).

To further elucidate the mechanism by which Talin-1 exerts its function, we explored downstream signaling pathways. Analysis of previous transcriptomic data revealed a significant difference in the PI3K-AKT pathway in the bacterial group (**Figure 2J**). Subsequently, PI3K-AKT activators and inhibitors were used for Western blot analysis to verify the effectiveness of pathway modulation (**Figure 6A**).We thus first validated the relationship between Talin-1 and the PI3K-AKT pathway. Following Talin-1 knockdown, phosphorylation levels of PI3K-AKT components were markedly decreased, indicating that Talin-1 activates the PI3K-AKT signaling cascade (**Figure 6C**). Next, we employed a PI3K-AKT inhibitor to investigate the pathway’s involvement in BMSCs osteogenesis. Inhibition of the PI3K-AKT pathway resulted in a significant restoration of osteogenic gene expression in BMSCs (**Figure 6E**). Western blot analyses further confirmed that PI3K-AKT inhibition notably rescued the bacterium-induced suppression of osteogenic protein expression (**Figure 6F**). These findings suggest that bacterial-induced activation of the PI3K-AKT pathway is critical for the attenuation of osteogenesis, while pathway inhibition reverses this effect. To validate the regulatory link between Talin-1 and PI3K-AKT, we treated Talin-1 knockdown BMSCs with a PI3K-AKT activator. Activation of PI3K-AKT signaling reversed the reduction in osteogenic gene and protein expression caused by Talin-1 deficiency (**Figures 6H–J**).

### 3.6 UM-164 blocks talin-1 binding, reducing phosphorylation & bacterial uptake, restoring BMSCs osteogenesis

**Figure 7:**
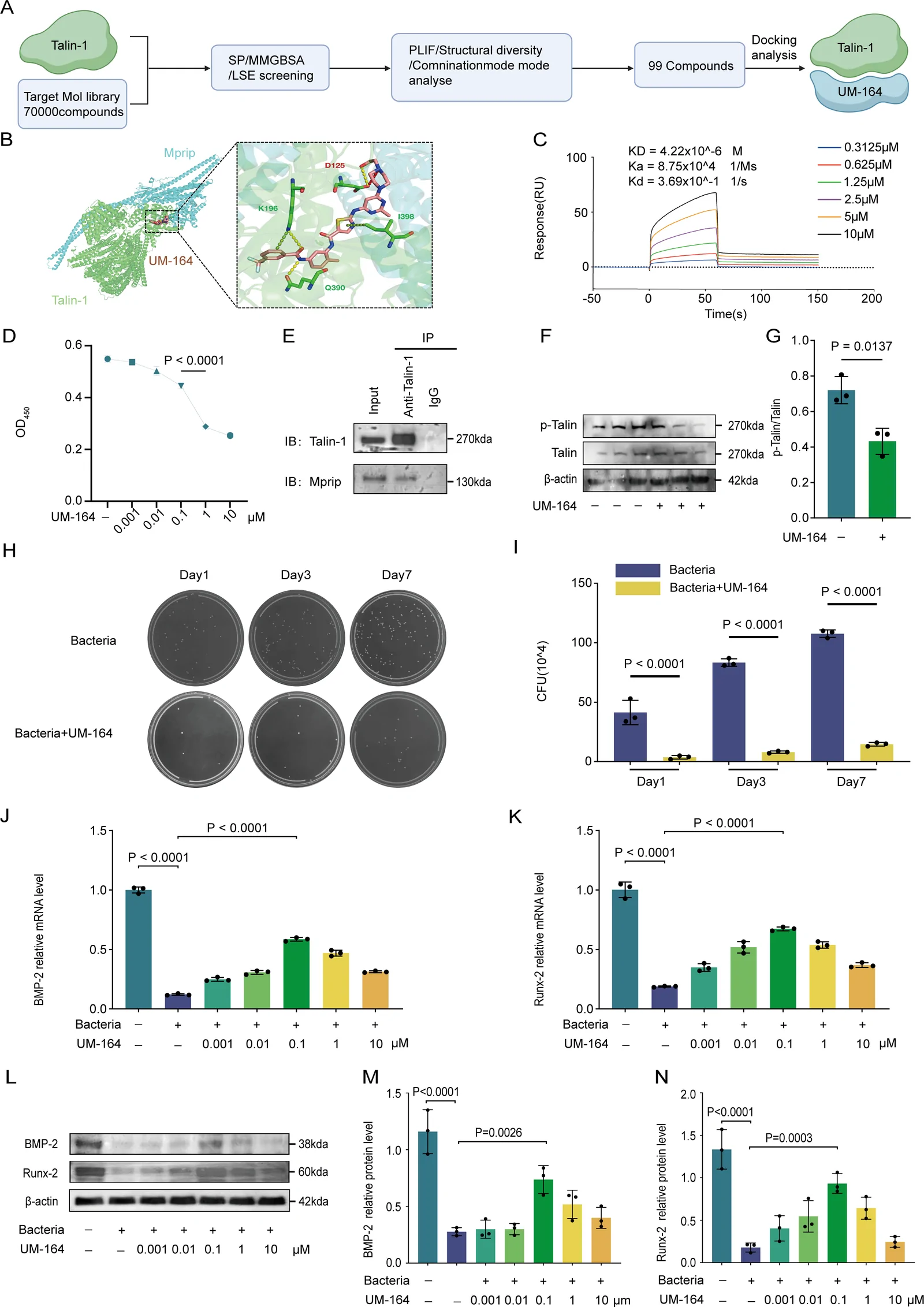
UM-164 blocks bacterial internalization and promotes bone formation by inhibiting talin-1 phosphorylation. **(A)** Virtual screening of small-molecule compounds binding to Talin-1. **(B)** Molecular docking simulation of UM-164 with Talin-1. **(C)** SPR assay evaluating the interaction (KD: dissociation constant, which reflects the affinity of the analyte to the target. The smaller the value, the stronger the affinity; Ka: association rate constant, which represents the speed of intermolecular binding. The larger the value, the faster the binding; Kd: dissociation rate constant, which represents the speed of intermolecular dissociation. The larger the value, the faster the dissociation). **(D)** CCK-8 cell viability assay performed on cells treated with UM-164 at concentrations of 0, 0.001, 0.01, 0.1, 1 and 10 μM. Cell viability decreased significantly as the concentration increased from 0.1 μM to 1 μM. **(E)** UM-164 treatment decreased the binding affinity between talin-1 and Mprip, as revealed by Co-IP assay. **(F, G)** Western blot analysis showing that UM-164 treatment decreases the phosphorylation level of Talin-1. **(H, I)** Intracellular bacterial CFU assays and statistical analysis comparing untreated and UM-164-treated cells (0.1 μM; labeled as “UM-164” in panel I) at 1/3/7 days post-infection. **(J, K)** RT-qPCR analysis of BMP-2 and Runx-2 expression levels following treatment with different concentrations of UM-164 (0, 0.001, 0.01, 0.1, 1 and 10 μM). **(L–N)** Western blotting and quantitative analysis of BMP-2 and Runx-2 protein levels after treatment with varying concentrations of UM-164 (0, 0.001, 0.01, 0.1, 1and 10 μM) (n = 3).

A small molecule was identified that targets the Mprip-Talin-1 interaction at residue D125, thereby inhibiting Talin-1 phosphorylation, suppressing bacterial invasion, and mitigating its detrimental effects on osteogenic differentiation. Initially, we conducted an in silico drug screening targeting Talin-1, evaluating 70,000 compounds from a small-molecule library (**Figure 7A**). Using AlphaFold3, we modeled the binding of these compounds to Talin-1, identifying UM-164 as a candidate that specifically binds to Talin-1 at D125 (**Figure 7B**). SPR was performed to validate the binding affinity between UM-164 and Talin-1, revealing a strong interaction with a dissociation constant (KD) of 4.22 × 10–^6^ M (**Figure 7C**). CCK-8 assays (**Figure 7D**), treatment with 0.1 μM UM-164 reduced the binding of Talin-1 to Mprip, as revealed by Co-IP assay (**Figure 7E**). Treatment with 0.1 μM UM-164 led to a significant decrease in Talin-1 phosphorylation relative to the control group (**Figures 7F, G**). At this concentration, bacterial colony formation assays further demonstrated a significant reduction in intracellular bacterial load after 1/3/7 days of UM-164 treatment (**Figures 7H, I**). To evaluate the impact of UM-164 on osteogenesis and cytotoxicity, qPCR analysis of osteogenic marker genes (**Figures 7I–K**), and western blotting for osteogenic protein expression (**Figure 7L**) were conducted. The concentration of 0.1 μM was identified as optimal, showing maximal restoration of osteogenic potential with minimal cytotoxicity (**Additional File: Supplementary Figure 3**).

### 3.7 UM-164 enhances antibiotic efficacy in murine osteomyelitis

**Figure 8:**
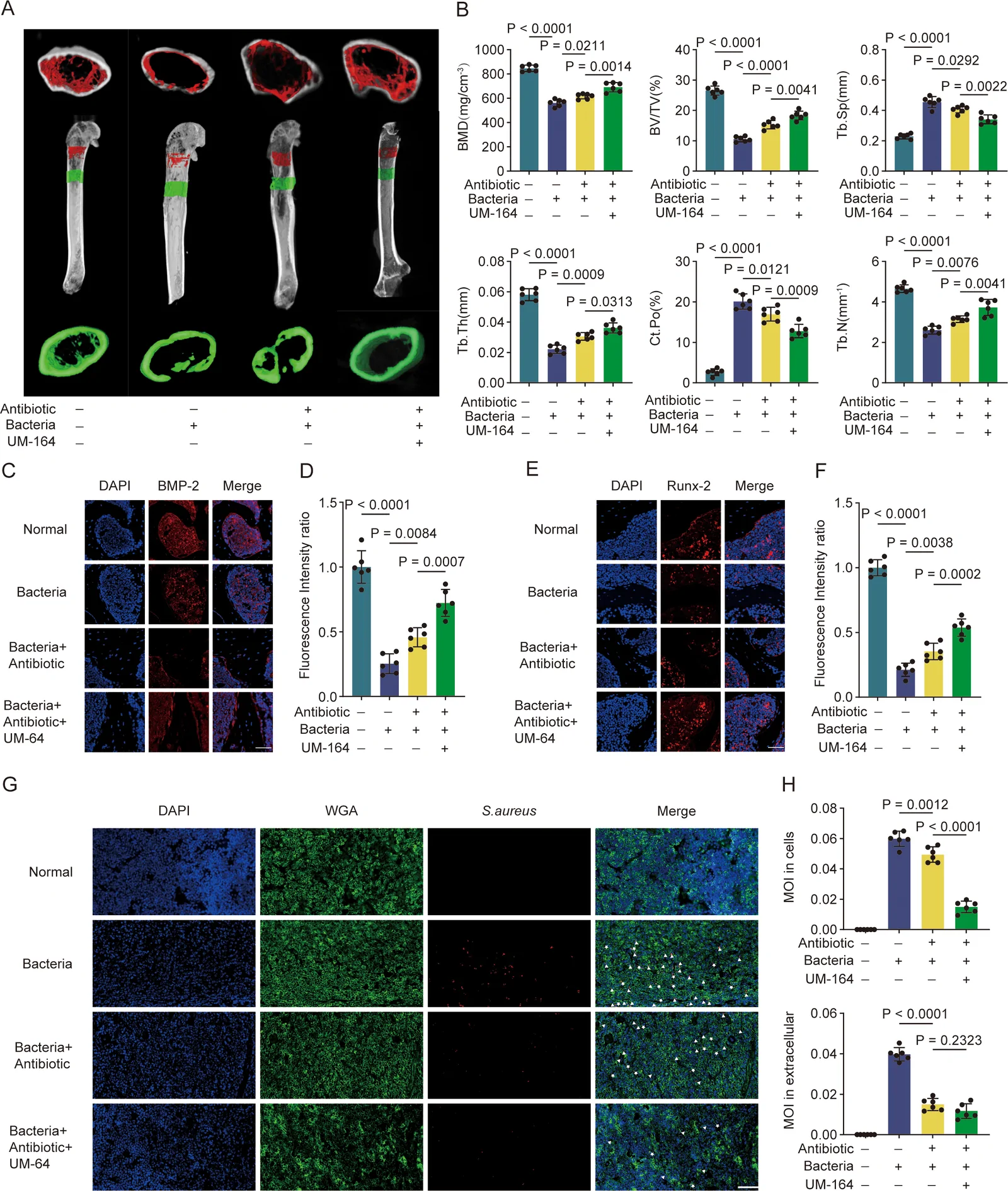
UM-164 blocks bacterial uptake and restores bone formation *in vivo*. **(A)** Micro-CT imaging of femurs from the four experimental groups: control, bacterial infection, antibiotic treatment, and antibiotic + drug treatment. **(B)** quantitative analysis of micro-CT derived trabecular and cortical bone parameters: BMD, BV/TV, Tb.Sp, Tb.Th, Ct.Po, and Tb.N. **(C, D)** Immunofluorescence staining of BMP-2 in the femur, along with quantitative analysis (original magnification ×63; scale bar = 50 µm; n = 6). **(E, F)** Immunofluorescence staining of Runx-2 in the femur, along with quantitative analysis. **(G)** Histological staining of *S. aureus* in the femurs of mice across different groups: normal, bacterial infection, antibiotic treatment, and antibiotic + UM-164 treatment. **(H)** Quantitative analysis of intra- and extracellular bacterial loads, as well as intra- and extracellular bacteria-to-host cell ratios.

Based on the aforementioned experiments, our results demonstrate that UM-164 effectively inhibits Talin-1 phosphorylation *in vitro*, thereby reducing bacterial invasion and enhancing the bactericidal efficacy of antibiotics, ultimately improving therapeutic outcomes. Subsequently, we conducted *in vivo* studies with the following groups: control, bacterial infection, antibiotic treatment, and combined antibiotic plus UM-164 treatment. The results showed that, compared to antibiotic monotherapy, the combination therapy significantly improved bone parameters in mice (**Figures 8A, B**). Immunofluorescence staining further revealed that the expression levels of osteogenic markers BMP-2 and Runx-2 were markedly higher in the combination group than in the antibiotic-only group (**Figures 8C–F**). Additionally, *S. aureus* staining in the femoral bones indicated a significant reduction in intracellular bacteria following UM-164 administration, with no significant difference observed in extracellular bacterial counts (**Figures 8G, H**). H&E staining of major organs (heart, liver, spleen, lung, kidney) revealed no significant toxicity before and after drug treatment (**Additional File: Supplementary Figure 4**).

## 4 Discussion

Current clinical management of osteomyelitis was primarily based on radical debridement and systemic antibiotics. However, therapeutic efficacy was often limited by poor penetration of antibiotics into host cells, resulting in persistent intracellular bacterial reservoirs and relapse (Sawaed et al., 2024; Jia et al., 2023). Although extensive research has been conducted on inflammatory cascades, local delivery systems, and novel antimicrobial agents, the host-pathogen interplay-particularly regarding intracellular invasion and its impact on bone regeneration-has been less extensively explored (Lu et al., 2024).

In this study, both *in vitro* and *in vivo* approaches were employed to investigate bacterial invasion mechanisms. Antibiotic treatment alone partially restored bone microarchitecture but failed to eliminate intracellular bacteria, resulting in incomplete recovery of osteogenesis. Transcriptomic, proteomic, and phosphoproteomic profiling revealed significant enrichment of phosphorylation-related enzymes and increased focal adhesion signaling, particularly via Fak, in infected cells (Marhuenda et al., 2025; Kanchanawong and Calderwood, 2023). Integrated multi-omics analyses further identified Talin-1 phosphorylation as a central mechanism through which bacteria hijack host cytoskeletal machinery, promoting internalization and repressing osteogenic transcription (Wu et al., 2022).

Analysis of multi-omics data revealed that the pathogen-induced structural recruitment of Mprip to Talin-1 D125 acts as a prerequisite for its subsequent phosphorylation. This modification cascade was found to disrupt focal adhesion dynamics and mechanotransduction, resulting in suppressed osteogenic differentiation via sustained activation of the PI3K-AKT pathway. Prevention of Talin-1 phosphorylation was shown to block bacterial internalization and restore osteogenic potential, underscoring the pivotal role of this modification in linking infection to impaired bone formation. These findings provide a mechanistic rationale for the development of host-directed therapeutic strategies targeting Talin-1 phosphorylation. It is worth noting that the involvement of Talin-1 in bacterial internalization is not restricted to *S. aureus* infection in bone-related cells. Indeed, Talin-1 represents a well-conserved host target frequently hijacked by diverse pathogens across various tissue types. For instance, in epithelial cells, *Salmonella enterica* recruits Talin-1 to trigger localized actin remodeling and membrane ruffling required for its engulfment (Finlay et al., 1991). Similarly, *Chlamydia trachomatis* leverages the integrin-Talin-1 focal adhesion complex as a critical entry portal to drive its host cell invasion (Stallmann and Hegemann, 2016). In this broader scientific context, our findings further highlight that Talin-1 serves as a universal mechanical and structural hub utilized by multiple bacteria to establish intracellular reservoirs.

To validate these mechanisms governing *S. aureus* evasion, various *in vitro* intracellular infection methodologies have been historically documented. Traditional assays often rely on brief, static co-cultures followed by immediate lysis to count intracellular colony-forming units or analyze acute signaling wavefronts (Hamza and Li, 2014). While effective for observing initial cellular entry, these conventional methods fail to replicate the chronic persistence phase of osteomyelitis. In contrast, our established “2-hour acute infection followed by a 24-hour (or 1, 3, 7 days) antibiotic suppression” paradigm offers significant superiorities. By utilizing a high-concentration combination of lysostaphin and gentamicin, our method achieves absolute elimination of extracellular and surface-adhered planktonic bacteria without inducing host cell cytotoxicity (Josse et al., 2015). More importantly, extending the antibiotic suppression phase up to 7 days provides a unique longitudinal window to observe the sustained, chronic metabolic and phenotypic shifts inside primary BMSCs. This prolonged containment strategy successfully decouples the downstream skeletal signaling cascades from the confounding artifacts of rapid extracellular bacterial overgrowth, offering a far more accurate pathological mimicry of the persistent intracellular bacterial reservoir characteristic of clinical chronic osteomyelitis (Collet et al., 2025).

Furthermore, our transcriptomic analysis and experimental validation identified the PI3K-AKT pathway as a significantly differentially regulated axis during *S. aureus* infection. In the pathological context of osteomyelitis, the PI3K-AKT signaling cascade serves as a central hub integrating mechanical stress, cellular survival, and inflammatory responses. Emerging evidence indicates that *S. aureus* frequently hijacks or disrupts the host PI3K-AKT axis upon internalization to modulate host cell processes. Specifically, the activation or alteration of PI3K-AKT signaling can suppress the autophagic clearance of intracellular pathogens, thereby promoting a permissive intracellular niche for bacterial survival (Sheng et al., 2026). Concurrently, in the skeletal microenvironment, dysregulated PI3K-AKT signaling heavily impairs downstream osteogenic transcription factors, directly contributing to the inhibition of BMSC differentiation and the exacerbation of inflammatory bone destruction (Ming et al., 2025). Mechanistically, our findings connect this pathway’s disruption to the physical Mprip-Talin-1 structural interface, demonstrating that targeted modulation of this mechanosensitive upstream axis can rescue the downstream PI3K-AKT imbalance, thereby restoring osteogenic potential while enhancing the clearance of the hidden intracellular bacterial pool.

Building on these mechanistic insights, UM-164 was identified as a small-molecule inhibitor targeting the critical D125 residue of Talin-1. The Mprip-Talin-1 interaction was effectively disrupted by UM-164, resulting in reduced Talin-1 phosphorylation, diminished intracellular bacterial load, and restored osteogenic differentiation *in vitro*. *In vivo*, intracellular bacterial levels were decreased by UM-164 monotherapy, while combination with systemic antibiotics led to a significant improvement in bone microarchitecture. This host-directed strategy not only enhances bacterial clearance but also preserves osteogenic function, providing a pathogen-agnostic approach that may limit the emergence of antibiotic resistance. This conceptual strategy aligns perfectly with current clinical paradigms, where the critical value of targeting intracellular bacteria is supported by robust clinical cohort data and randomized trials, demonstrating that combining cell-penetrating antibiotics, such as rifampin, significantly reduces recurrence rates and improves infection clearance in patients with chronic *S. aureus* osteomyelitis (Sendi and Zimmerli, 2012).

Despite these promising results, several limitations should be acknowledged. First, the study was conducted primarily in rodent models, which may not fully replicate human osteomyelitis. Large animal studies are required to evaluate translational applicability (Long et al., 2025; Abe et al., 2023). Second, although preliminary toxicity assessments of UM-164 indicated short-term safety, long-term evaluations-including hepatic, renal, and immunomodulatory effects-are needed (Kang et al., 2024; Haacke et al., 2025). Third, *S. aureus* was employed as the model pathogen, and the conservation of Talin-1 phosphorylation-mediated invasion across other clinically relevant intracellular pathogens has yet to be determined. Future research should aim to validate these findings across diverse pathogens, optimize delivery strategies, and explore the potential for clinical translation. Fourth, although AlphaFold3-based structural modeling and site-directed mutagenesis coupled with co-IP and pull-down assays identified the D125 residue as the primary interface mediating the interaction between Mprip and Talin-1, a comprehensive cell-level rescue assay utilizing Talin-1 wild-type and D125 mutant constructs under an endogenous Talin-1-deficient background was not fully accomplished. Due to the inherent transfection resistance and high sensitivity of primary BMSCs to sequential genetic manipulation, demonstrating the precise functional recovery of Talin-1 phosphorylation kinetics and downstream osteogenic signaling remains technically challenging in this primary cell model. Future efforts utilizing CRISPR-mediated locus-specific knock-in models or highly efficient viral transduction systems in stable cell lines will be instrumental to definitively map the causal chain connecting the explicit D125 structural interface to the inhibition of bacterial internalization.

In conclusion, a previously unrecognized mechanism-the specific structural binding of Mprip to Talin-1 and its subsequent phosphorylation cascade-was revealed to underlie bacterial internalization and suppression of osteogenesis. Targeting this axis with UM-164 is suggested as a promising host-directed therapeutic strategy, combining enhanced bacterial clearance with restoration of bone homeostasis, and demonstrating strong translational potential for the treatment of persistent osteomyelitis.

## 5 Conclusions

Our study reveals that Talin-1 phosphorylation is a central mechanism driving bacterial invasion and disruption of bone homeostasis in osteomyelitis. Targeting this process with the dual-action inhibitor UM-164 effectively blocks bacterial internalization, synergizes with antibiotics, and restores osteogenic function, highlighting a promising host-directed therapeutic strategy. These findings advance our understanding of osteomyelitis pathogenesis and offer a strong translational potential for clinical application in treating persistent bone infections.

## Data Availability

The datasets presented in this study can be found in online repositories. The names of the repository/repositories and accession number(s) can be found in the article/**Supplementary Material**.
