# Supplementary material for: Enhancement of antibiotic therapy in osteomyelitis by inhibiting talin-1-mediated bacterial internalization
Source: Front Cell Infect Microbiol. 2026 Aug 6;16:1823065. doi: 10.3389/fcimb.2026.1823065 (PMC13489879; doi:10.3389/fcimb.2026.1823065)
Supplement: Supplementary file 1 [file Table1.docx]

**Supplementary Information**

**Title: Enhancement of antibiotic therapy in osteomyelitis by inhibiting Talin-1-mediated bacterial internalization**

**Supplementary Figures:**

**Fig. S1** (**A)** Principal component analysis (PCA) plot. (**B)** Top 10 differentially expressed genes.

**Fig. S2** Effect of *S. aureus* infection and Talin-1 knockdown on BMSC proliferation. Cell proliferation curves of primary BMSCs in the Normal, Bacteria, Bacteria+si-NC, and Bacteria+si-Talin-1 groups evaluated by CCK-8 assay at indicated time points (1, 3, and 7 days). Data are presented as mean ± SD. Statistical significance was determined by two-way ANOVA. *P* < 0.0001 versus the Normal group.

**Fig. S3** After treatment with UM-164 (0.1 μm/ml). **(A)** RT-qPCR analysis of BMP-2 and Runx-2 in Normal, Bacteria, and Bacteria + UM-164 groups. **(B-C)** Western blot analysis and quantification of Control, Bacteria, and Bacteria + UM-164 groups (*n* = 3).

**Fig. S4** Comparative analysis of H&E staining in the heart, liver, spleen, lung, and kidney of mice after one month of UM-164 treatment (scale bar = 200 µm).

**Table S1** Primer sequences used in RT-qPCR

| Gene | Forward | Reverse |
| --- | --- | --- |
| BMP-2 | CTGCGGTCTCCTAAAGGTCG | ATGCTGAGCAGCCTCAACTC |
| Runx-2 | GGGAACCAAGAAGGCACAGA | ACTTGGTGCAGAGTTCAGGG |
| Alpl | ACAGCAGTTGGTAGCTTCCTT | TTAATTGACGTTCCGATCCTGAG |
| Cola1 | CGATGGATTCCCGTTCGAGT | CGATCTCGTTGGATCCCTGG |
| β-actin | TATAAAACCCGGCGGCGCA | TCATCCATGGCGAACTGGTG |

**Table S2** Sequences of siRNA used in cell transfection

| Gene | Forward | Reverse |
| --- | --- | --- |
| Tln1-1 | GGAUGUAGCUGGUGGACUATT | UAGUCCACCAGCUACAUCCTT |
| Tln1-2 | GCCAAAGAGACGUGGAUAATT | UUAUCCACGUCUCUUUGGCTT |
| Tln1-3 | CAGUGAAGGCUGUGGAAGATT | UCUUCCACAGCCUUCACUGTT |
| Mprip-1 | GGAGAUGGAACGAGAGUUATT | UAACUCUCGUUCCAUCUCCTT |
| Mprip-2 | UGACCUAGAUGGAGAAAUUTT | AAUUUCUCCAUCUAGGUCATT |
| Mprip-3 | GAAGAAGAUUGAAGACCUATT | UAGGUCUUCAAUCUUCUUCTT |
